# Insights Into Meningioma Visibility on Arterial Spin Labeling MRI: Location Outweighs Size

**DOI:** 10.7759/cureus.40204

**Published:** 2023-06-10

**Authors:** Emilian Kalchev

**Affiliations:** 1 Diagnostic Imaging, St. Marina University Hospital, Varna, BGR

**Keywords:** perfusion imaging, tumour location, tumour size, magnetic resonance imaging (mri), arterial spin labelling (asl), meningiomas

## Abstract

Background

Arterial Spin Labeling (ASL) MRI is a non-invasive imaging technique with potential applications for assessing meningiomas. This retrospective study aimed to investigate the impact of tumor location, size, age, and sex on the ASL visibility of meningiomas.

Methods

We retrospectively analysed 40 patients with meningiomas, who underwent 3 Tesla MRI examinations using a three-dimensional (3D) pulsed ASL technique. Tumor location was categorized as around the skull base or elsewhere, and size was determined by the area in the transverse plane.

Results

Our findings revealed that meningiomas around the skull base were significantly more likely to be ASL-visible compared to those located elsewhere (p < 0.001), whereas tumor size, age, and sex did not show a significant correlation with ASL visibility. This observation suggests that tumor location is a critical factor in determining the visibility of meningiomas on ASL MRI.

Conclusion

The results contribute to a better understanding of ASL visibility in meningiomas, highlighting the importance of tumor location over size. Further research, including larger cohorts and additional factors, such as histological variants, is needed to expand upon these findings and explore their clinical implications.

## Introduction

Meningiomas are the most common primary intracranial tumors, accounting for approximately 30% of all such tumors [1]. They arise from the arachnoid cells of the meninges and can occur at various locations within the central nervous system [2]. The clinical management of meningiomas relies on accurate diagnosis and characterization, which often involves multiple imaging techniques, including magnetic resonance imaging (MRI) [3].

Arterial Spin Labelling (ASL) is a non-invasive MRI perfusion technique that utilizes magnetically labeled arterial blood water as an endogenous tracer to measure cerebral blood flow [4]. It offers several advantages over other perfusion techniques, such as the absence of ionizing radiation and the use of exogenous contrast agents, making it particularly suitable for patients with renal impairment or contrast allergies [5].

Although ASL has been increasingly applied to evaluate various brain tumors, including meningiomas [6,7], the impact of tumor location, size, age, and sex on ASL visibility has not been comprehensively explored. A better understanding of these factors may help refine the utility of ASL in the evaluation of meningiomas and inform clinical decision-making.

In this study, we retrospectively analyzed 40 patients with meningiomas who underwent 3 Tesla MRI examinations using a three-dimensional (3D) pulsed ASL technique. Our primary aim was to determine whether the visibility of meningiomas on ASL MRI is dependent on tumor location or size. We also adjusted our analysis for age and sex to explore the potential influence of these variables on ASL visibility.

## Materials and methods

Study design and patient population

In this retrospective observational study, we analyzed data from 40 patients diagnosed with meningiomas, who underwent magnetic resonance imaging (MRI) examinations at St. Marina University Hospital, Varna, Bulgaria, between January 2022 and December 2022. Exclusion criteria were: poor image quality due to motion or other artifacts or incomplete MRI protocol. This study involved an anonymized and de-identified dataset. As per institutional guidelines, this type of study did not necessitate the acquisition of ethics committee approval.

MRI protocol

The MRI examinations were conducted using a 3 Tesla MRI scanner (Magnetom Verio, Siemens Healthcare), deploying a standardized protocol, which included 3D pulsed ASL sequences for qualitative assessment of perfusion. The ASL sequence parameters were as follows: repetition time (TR) = 8000 ms, echo time (TE) = 16.6 ms, inversion time (TI) = 1990 ms, and a voxel size of 3 x 3 x 3 mm³. The protocol also encompassed pre- and post-contrast T1-weighted, T2-weighted, diffusion-weighted imaging (DWI) and fluid-attenuated inversion recovery (FLAIR) sequences. This comprehensive imaging protocol ensured a thorough evaluation of the meningiomas and their surrounding structures.

Image analysis

To ensure the accuracy and reliability of the data used, we implemented a stringent data validation process. Two experienced radiologists independently reviewed each case. Any discrepancies between their assessments were resolved through discussion and consensus. In cases where a consensus could not be reached, a third radiologist made the final decision. This process allowed the elimination of potential errors or inconsistencies in the data, further bolstering the reliability of the findings.

The tumor size (in mm²) was measured on the largest cross-sectional area visible on the post-contrast T1-weighted images. The tumor location was classified into two categories: those around the skull base and those located elsewhere in the brain.

The ASL visibility of each tumor was evaluated qualitatively. Tumors were considered visible on ASL if they displayed increased signal intensity relative to the surrounding brain parenchyma on the ASL images.

To maintain patient anonymity, all identifiers were meticulously removed from the imaging data prior to the analysis. The research design was purely observational, involved no patient interaction, and all the data were processed in aggregate.

Statistical analysis

For a comprehensive understanding of the data, descriptive statistics were calculated for all variables. Logistic regression was employed to assess the relationships between ASL visibility (dependent variable) and each independent variable (tumor location, size, age, and sex). To account for the effect of confounding variables, the regression was adjusted for the remaining variables. To estimate the strength of the associations, odds ratios (ORs) and 95% confidence intervals (CIs) were calculated for each variable. A p-value of <0.05 was considered statistically significant, indicating a meaningful relationship. All statistical analyses were executed using the most recent version of SPSS (version 29; IBM Corp., Armonk, NY, USA).

## Results

Patient characteristics

The study population consisted of 40 patients with meningiomas, with a mean age of 56.90 years (range: 15-80 years, SD: 13.12) and a male-to-female ratio of 0.3. The mean tumour size was 381.75 mm^2^ (range: 22-1797 mm^2^, SD: 449.04). In total, 20 tumours (50%) were located around the skull base, and 20 (50%) were located elsewhere. ASL visibility was observed in 21 patients (52.5%), while 19 patients (47.5%) had tumours that were invisible on ASL. More specifically, when looking at tumor location, out of the meningiomas located around the skull base, 90% (18 out of 20) were visible on ASL. However, for meningiomas located elsewhere, only 15% (three out of 20) were visible on ASL.

Associations between ASL visibility and independent variables

**Figure 1:**
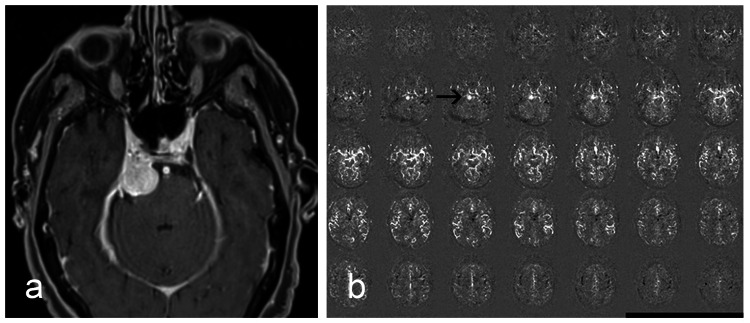
Meningioma in the right cerebellopontine angle. (a) Contrast-enhanced T1-weighted image. (b) ASL mosaic images with increased signal in the area of the tumour (black arrow). Meningioma is clearly visible despite the global ASL perfusion signal decline in the brain.

**Figure 2:**
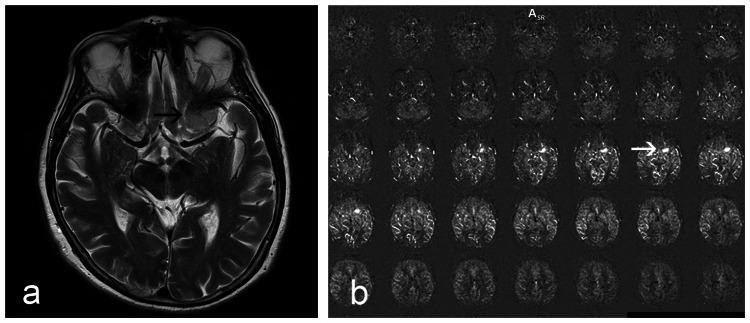
Meningioma around the left clinoid process. (a) T2-weighted image (black arrow). (b) Meningioma visible on ASL images (white arrow).

**Figure 3:**
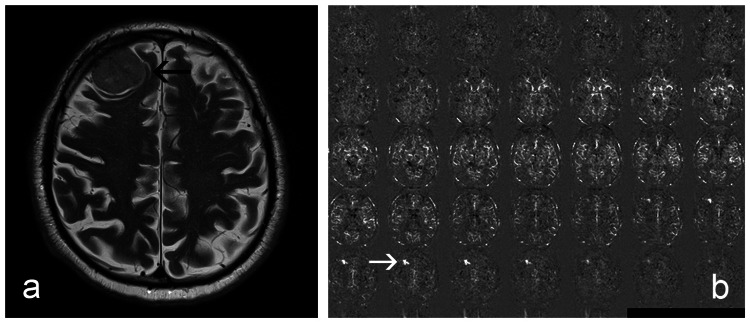
Meningioma adjacent to the frontal bone on the right. (a) T2-weighted image with the black arrow indicating tumour. (b) ASL images show increased signals in some areas (white arrow).

**Figure 4:**
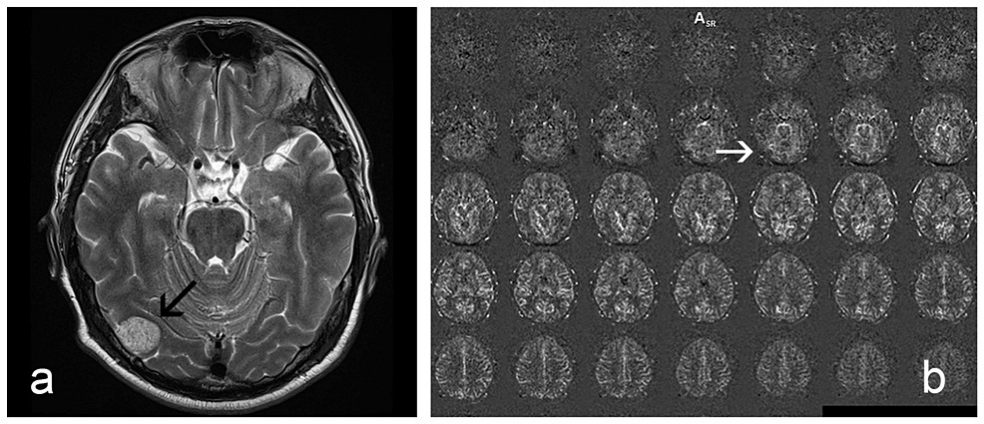
Meningioma in the right occipital region. (a) T2-weighted image displaying the tumor (black arrow). (b) Corresponding ASL images reveal no signal enhancement in the tumor area (white arrow).

Binary logistic regression analysis revealed a significant association between tumour location and ASL visibility. Meningiomas located around the skull base were significantly more likely to be visible on ASL compared to those located elsewhere (OR = 54.081, 95% CI: 5.92-494.57, p < 0.001). (Figures 1-4)

No significant associations were found between ASL visibility and tumour size (OR = 1.001, 95% CI: 0.997-1.005, p = 0.524), patient age (OR = 0.985, 95% CI: 0.908-1.068, p = 0.721), or sex (OR = 1.481, 95% CI: 0.135-16.235, p = 0.748).

These results indicate that the location of meningiomas, specifically around the skull base, is significantly associated with their visibility on ASL MRI, while tumour size, patient age, and sex do not appear to have a significant impact on ASL visibility in this population.

## Discussion

This retrospective investigation centered on assessing the visibility of meningiomas on arterial spin labeling (ASL) magnetic resonance imaging (MRI), with particular attention given to factors such as tumor location, size, patient age, and sex. Our results suggest that tumor location, specifically the presence of meningiomas around the skull base, has a more pronounced effect on ASL visibility than tumor size.

The observed heightened ASL visibility of meningiomas situated around the skull base could potentially be explained by multiple factors. One plausible reason could be the increased vascularity frequently associated with skull base meningiomas compared to those located elsewhere [8]. Higher vascularity would result in higher perfusion, thus causing increased ASL signal intensity [9,10]. In addition to that, skull base meningiomas' proximity to large blood vessels could contribute to their enhanced visibility on ASL. The localized increase in blood flow due to adjacent major vessels might influence the ASL signal and hence the visibility of these tumors [11].

Moreover, while tumor size is an intuitive parameter to consider in assessing tumor visibility, our study surprisingly found no significant correlation between tumor size and ASL visibility when adjusting for other variables such as age, sex, and tumor location. This finding adds depth to our understanding of the complexities involved in ASL signal variation and its interpretation in MRI imaging of meningiomas [12-14].

However, as with all scientific research, our study was not without limitations. The most noteworthy of these include a relatively small sample size, which may have constrained the statistical power to detect significant associations, especially with variables like tumor size. This could potentially mask some subtle associations and warrants caution while interpreting the results.

Furthermore, the study design being retrospective could introduce selection bias, as the included patients might not be entirely representative of all patients with meningiomas. Another limitation is the ASL technique's inherent qualitative nature, which could potentially limit the accuracy of ASL visibility evaluation.

We also did not account for other potentially influential tumor characteristics, such as histopathological subtype, vascularity, degree of calcification, or the presence of cystic components, each of which might impact ASL visibility. Incorporation of these factors into the analysis would have provided a more holistic understanding of the determinants of ASL visibility in meningiomas [15,16].

Despite these potential limitations, our study remains a valuable contribution to the existing body of knowledge concerning meningioma visibility on ASL MRI. The study underscores the critical role of tumor location when assessing meningiomas with ASL, a finding that is of considerable relevance to radiologists and neurosurgeons alike.

Looking ahead, future studies should focus on validating these findings with larger cohorts and incorporating quantitative ASL techniques. Quantitative ASL could potentially provide more precise and objective data regarding tumor perfusion, which would be valuable in understanding the clinical implications of these factors in the management of meningiomas. Additionally, future research could also explore the influence of other tumor characteristics and histopathological subtypes on ASL visibility, thereby providing a more comprehensive perspective on this important topic.

This study underlines the promise of ASL MRI in assessing meningiomas and urges further research in this area, potentially opening new paths for better clinical decision-making and patient management in the future.

## Conclusions

In conclusion, our study demonstrated that the location of meningiomas, particularly those around the skull base, is significantly associated with their visibility on ASL MRI. This finding may have important implications for the optimisation of imaging protocols and clinical management strategies for meningiomas. However, no significant associations were found between ASL visibility and tumour size, patient age, or sex, suggesting that these factors may have a limited impact on the diagnostic performance of ASL for meningiomas.

Our results also provided insights into potential areas of interest for future research, such as the possible reasons for better ASL visibility of meningiomas around the skull base, possible threshold size for ASL visibility, and the potential for limiting the use of intravenous (IV) contrast when examining patients with meningiomas. Further studies incorporating larger patient cohorts, quantitative ASL measures, and a more comprehensive evaluation of tumour characteristics are needed to enhance our understanding of the factors influencing ASL visibility in meningiomas and to improve the clinical utility of this imaging modality.
